# Summer Savory (*Satureja hortensis* L.) Extract as Natural Feed Additive in Broilers: Effects on Growth, Plasma Constituents, Immune Response, and Ileal Microflora

**DOI:** 10.3390/ani9030087

**Published:** 2019-03-11

**Authors:** Sajjad Movahhedkhah, Behrouz Rasouli, Alireza Seidavi, Domenico Mazzei, Vito Laudadio, Vincenzo Tufarelli

**Affiliations:** 1 Department of Animal Science, Rasht Branch, Islamic Azad University, Rasht 41335-3516, Iran; movahedkhah.farzad@gmail.com (S.M.); brasooli@gmail.com (B.R.); alirezaseidavi@iaurasht.ac.ir (A.S.); 2 Department of DETO, Section of Veterinary Science and Animal Production, University of Bari ‘Aldo Moro’, Valenzano, 70010 Bari, Italy; Domenico.mazzei@uniba.it (D.M.); vito.laudadio@uniba.it (V.L.)

**Keywords:** broiler, feed additives, summer savory, herbs, growth, health

## Abstract

**Simple Summary:**

The growth-promoting effect of many herbs and their extracts in poultry has been reported in literature. Therefore, the objective of this feeding trial was to determine the effect of different levels of summer savory (*Satureja hortensis* L.) extract in diet on broilers performance, immune response, hematology, and microbiota. Based on findings, dietary supplementation with summer savory extract, as natural feed additive, sustained growth traits and improved the feed efficiency and health status of broilers.

**Abstract:**

This study investigated the effects of summer savory (*Satureja hortensis* L.) extract (SSE) on growth, plasma constituents, immune response, and gut microbiota of broiler chickens. A total of 300 day-old broiler chicks were randomly assigned to five dietary treatments containing five replicates of 12 birds each. The treatments consisted of a controldiet without feed additive and experimental diets supplemented with four levels of SSE (100, 200, 300, and 400 mg/kg, respectively). Results showed no significant effect of SSE supplementation on broiler body weight gain (*p* > 0.05), but feed conversion ratio was significantly (*p* < 0.05) improved when fed 400 mg/kg SSE compared to control. Most of the blood parameters and immune response criteria studied were improved (*p* < 0.05) by SSE supplementation. There was no dietary effect on Lactobacilli count (*p* > 0.05); conversely, *Escherichia coli* count was reduced and the Lactobacilli/*E. coli* ratio improved with SSE (*p* < 0.05). Based on our findings, it was concluded that supplementation of the diet with SSE up to 400 mg/kg sustained growth traits and improved the feed efficiency and health status of broilers. However, more research is needed on this subject in order to better understand the mode of action of the extract used.

## 1. Introduction

The gradual ban of antibiotics in animal feed due to their health hazards to both animal and human consumers of animal products has increased the research interest into natural products with antimicrobial activity [1,2]. The use of medicinal herbs and their extracts as natural growth promoters in poultry diets has been reported [3,4,5].

Savory (*Satureja hortensis* L.) is an annual herbaceous aromatic and medicinal plant belonging to the Lamiaceae family. Savory is a good source of essential oils, mainly carvacrol and thymol, which have several health benefits including anti-inflammatory [6,7], antioxidant [8], antibacterial [8,9], and antifungal [10] activities in animals and humans. Thus, it may be worth investigating the effect of summer savory extract on poultry production. 

Therefore, the present study tested the hypothesis that dietary summer savory extract (SSE) could improve and sustain the growth performance, plasma constituents, immune response, and ileal microflora of broiler chickens during a 42-day production cycle.

## 2. Materials and Methods

### 2.1. Savory Extract

Savory plant aerial parts were harvested and sun-dried, and extract was prepared as previously described [11]. Briefly, dried savory was infused with boiling water at 100 °C (1 L water:200 g dry herb) for 10 min, then cooled at room temperature and strained to obtain summer savory extract (SSE). The extract was analyzed by gas chromatography and mass spectrometry [12] to contain 33 and 38 g/kg of carvacrol and thymol, respectively.

### 2.2. Animals, Diets, and Management

The study was conducted on a commercial poultry farm in Rasht, Iran. The experimental protocol was approved by the Animal Ethic Committee of the Islamic Azad University, and the experiment was conducted in accordance to the International Guidelines for Research involving animals (Directive No. 2010/63/EU). 

A total of 300 Ross 308 male broiler chicks were used in the feeding trial. Thermo-neutral ambient temperature was maintained in accordance to standard brooding practices and adapted to the birds rearing stages (Aviagen, Newbridge, Scotland, UK). Lighting was provided for 24 h on day 1, and 23 h per day thereafter. Broiler diets were formulated (Aviagen, Newbridge, Scotland, UK) for three growth periods (starter: 1–14 d; grower: 15–28 d; and finisher: 29–42 d) as mash form (Table 1). Birds were vaccinated against the infectious bronchitis (IBV; H120) at days 1 and 8, Gumboro (IBD071IR) at days 16 and 32, Newcastle (Lasota) at days 1 and 8, and avian influenza (H5) at day 1. Birds were allotted to 25 floor pens (1.0 × 2.0 m) containing 12 birds each. Broilers in five replicatepens received 0, 100, 200, 300, and 400 mg/kg SSE in a completely randomized design. Feed and water were provided ad libitum.

### 2.3. Growth Traits

Feed consumption was assessed by difference between the quantity fed and left over. Birds were weighed at the beginning of the experiment and end of each growth phase to determine the body weight gain. Feed conversion ratio (FCR) was calculated as the ratio of feed consumed to body weight gained.

### 2.4. Blood Metabolites and Hepatic Enzymes

At the end of the experiment (42 days), two birds were randomly selected from each replicate (eight birds per treatment) and fasted (4 h) and used for blood sampling. Blood samples (~5 mL/bird) were collected from wing vein into tubes containing 10 mg ethylene diaminetetra-acetic acid (EDTA) for plasma separation. Serum was separated by centrifugation (3000*g* × 10 min at room temperature) and stored at −20 °C until analyses. Parameters determined were as follows: glucose, triglycerides, uric acid, total cholesterol, high density lipoprotein (HDL), low density lipoprotein (LDL), HDL to LDL ratio, and alkaline phosphatase. Blood traits were determined based on standard protocols using commercial kits as reported in Nahavandinejad et al. [13]. 

### 2.5. Immune Competency

To evaluate the immunity, three birds per pen were challenged with sheep red blood cells (SRBC) twice, at days 13 and 24, and blood was sampled at days 22 and 38 for assessment of total antibody, IgG, and IgM production. For the SRBC challenge, 0.5 mL of a 10% suspension of SRBC in sterile phosphate buffered saline (PBS) solution (*v*/*v*) was inoculated under skin of breast. In each replicate, only two birds were tested. In these birds a pre-immune blood sample was collected. For the assessment of the immune parameters, blood samples (~2 mL) were collected from wing vein on the pre-scheduled days. The samples were centrifuged at 1500 rpm × 10 min and serum was harvested and stored at −20 °C until analysis. Response to the Newcastle lentogenic vaccine was assessed in blood twice, at days 15 and 26. Lyophilized vaccines (Razi Co., Tehran, Iran) were prepared with strains Hitchner B1, Lasota, and Clon 30, and administered on days 1, 35, and 42, respectively. Hemagglutination inhibition (HI) assays were used to determine the vaccine titres of Newcastle disease (ND) following the procedure described in previous trials [14,15]. On day 42, blood samples were collected from three randomly selected birds per replicate. Serum was separated by centrifugation (3000*g* × 15 min) and antibody titers against the infectious bursal disease (IBD) and infectious bronchitis (IB) viruses were measured using commercially available ELISA kits [3].

### 2.6. Ileal Microflora

Agar plates were streaked with ileal content and sent to the laboratory for determination of bacterial growth and colony counts [5]. Collecting tubes were weighted, wrapped in an aluminum sheet, and then autoclaved for 10 min. The culture media were prepared and poured into the Petri dish 24 h before sample collection. The MRS agar (de Man Rogosa Sharpe agar, 1.10660.500) was used to culture Lactobacilli, and Eosin Methylene Blue (EMB, 1.01347.0500) to culture *Escherichia coli* Collection tubes were weighed empty and with sample, and the weight of sample in each tube was calculated by recording the difference between the two values. Tubes were shaken for approximately 30 min. The action was performed for bacteria isolated from gastrointestinal contents and preparation of suspension. About 1 mL was removed from the suspension and added into 9 mL PBS in the other tube. Lactobacilli bacteria were incubated at 37 °C in anaerobic conditions for 72 h. A bacteria count was done using a colony counter and counts were reported as log of bacteria number per 1 g of sample.

### 2.7. Statistical Analysis

Data were analyzed using one-way ANOVA of GLM procedure (IBM SPSS Statistics software for Windows^®^) [16]. The pen was considered as the experimental unit for growth parameters, whereas individual bird data was considered for blood constituents, immune response, and gut microflora. The effect of SSE levels in diet was determined using orthogonal polynomials for linear and quadratic effects. Significant differences among group means were reported at *p* < 0.05. 

## 3. Results

The growth performance results are summarized in Table 2. During the initial growth phase (1–14 d), there were no effects on broilers feed intake and body weight gain (*p* > 0.05). In the grower phase (15–28 d), feed intake was quadratically reduced (*p* < 0.05) when broilers were fed more than 300 mg/kg SSE. None of the growth parameters evaluated were affected (*p* > 0.05) by dietary treatments during the finisher phase (29–42 d).

The combined growth performance (1–42 d) showed lower feed intake on 100 mg/kg SSE compared to the control (*p* < 0.05). Feed intake was not different (*p* > 0.05) among the control, 200, 300, and 400 mg/kg SSE and between the SSE groups. The FCR was improved linearly when fed 400 mg/kg diet (*p* < 0.05) compared to control and 300 mg/kg SSE. 

Serum glucose concentration (Table 3) showed the lowest value (linear, *p* < 0.05) at 200 and 300 mg/kg compared to 100 and 400 mg/kg SSE. Inclusion of SSE reduced serum uric acid concentration (quadratic, *p* < 0.05). Total cholesterol was lowered at 300 mg/kg and triglycerides concentration was reduced (quadratic, *p* < 0.05) at 200 and 300 mg/kg SSE in diet. Significantly lower HDL and higher LDL cholesterol and alkaline phosphatase values were observed in the control diet group (quadratic, *p* < 0.05). In addition, plasma LDL was also lowered (quadratic, *p* < 0.05) in groups on 300 and 400 mg/kg SSE compared to the control.

The immune response of the broilers (Table 4) showed a lower IgG at 400 mg/kg compared to 300 mg/kg SSE (28 d; linear and quadratic, *p* < 0.01). The IgG values were not different (*p* > 0.05) among control, 100, 200, and 300 mg/kg groups at 42 d. The IgM was not affected by treatments at 28 d (*p* > 0.05), but higher values were observed among SSE groups at 42 d (linear and quadratic, *p* < 0.01).

The anti-Newcastle disease hemagglutination-inhibition titre increased above 100 mg/kg SSE in diet (linear, *p* < 0.05 and quadratic, *p* < 0.01). The titre of IBD virus was not affected by the treatment (*p* > 0.05), but titre of IB virus was significantly increased by SSE supplementation (linear, *p* < 0.01). 

The ileal microflora count (Table 5) showed a significant reduction of *E. coli* in SSE-supplemented groups (quadratic, *p* < 0.01); the count of Lactobacilli was not affected by treatments (*p* > 0.05), but the ratio of Lactobacillito *E. coli* was significantly increased with SSE supplementation (linear and quadratic, *p* < 0.01) and maximized at 100 mg/kg SSE.

## 4. Discussion

The available published literature on the use of summer savory as feed supplement in poultry diet is scanty. In the present study, the inclusion of SSE did not affect broilers body weight gain and the pattern of feed intake could not be traced to any specific treatment effects. The pattern of weight gain observed in our study is in agreement with the report of Nobakht et al. [3] who fed summer savory powder to broiler chickens. Contrary to our results, however, the authors observed improved feed intake on savory-based diets compared to control, but feed conversion ratio was not affected by savory supplementation. We used savory extract in our investigation and not savory powder as in their study. The type of savory product and possible differences in composition may affect the growth performance in broilers. 

Essential oils are reported to have several beneficial functions in poultry diet, including increased feed intake [17,18], digestibility enhancement [19], secretion of digestive enzymes [17,18,19], and balancing of gut microbial ecosystem [20]. The improved performance observed with the addition of SSE could be due to these beneficial properties of essential oils in this herb. In a previous study, Tiihonen et al. [21] observed a significant improvement in feed efficiency when broilers fed thymol- and cinnamaldehyde-supplemented diets over the control. Several earlier studies have also reported improved bird performance with essential oils supplementation [17,18,19,22]. 

There is a current lack of published data on the effects of feeding of savory products on plasma constituents in broiler chickens. The pattern of serum constituents observed in this study may be speculatively linked with the digestive properties of essential oils in savory. Dietary supplementation of thymol was reported to significantly increase pancreatic activity in broilers [19]. Increased protein digestibility due to higher activity of pancreatic proteases could be a possible reason for lower uric acid concentration in the SSE-supplemented groups in our study. The cholesterolemic effect of essential oils [23,24] may be used to explain the significant reduction of cholesterol at 200 and 300 ppm SSE inclusion in diet as well as LDL at 300 and 400 mg/kg SSE. The only published report we came across on the effect of savory supplementation on the immune response of broilers [3] was not conclusive. From our results, the response of birds to most immunological parameters measured improved in a later stage of growth, suggesting that the immune response of broilers to SSE-supplemented diets is age-dependent and may be due to the antioxidants and essential oil contents of the used extract [25,26]. The significant reduction in *E. coli*, similarity to Lactobacilli counts, and the higher ratio of Lactobacillito coliforms suggests better gut health of the SSE-treated birds. This pattern of IBD and IB virus titres, *E. coli* and *Lactobacilli* counts observed may suggest a selective antimicrobial activity of savory extract. Moreover, the improved gut ecology may explain the increase in the antibody titers due to nutrient sparing effect. However, more studies are needed in this area.

## 5. Conclusions

In conclusion, the dietary supplementation with summer savory extract up to 400 mg/kg, as natural feed additive, sustained growth performance and improved the health status of broiler chickens. However, further investigation is recommended in order to establish a better understanding of the mode of action of the extract.

## Figures and Tables

**Table 1:** Composition of the experimental basal diets fed to broiler chickens.

Ingredients (g/kg, as Fed Basis)	Days		
	1–14 d	15–28 d	29–42 d
Corn	518.3	585.9	615.5
Soybean meal (44% crude protein)	426.8	356.5	318.6
Soybean oil	16.0	22.0	31.7
Limestone	12.7	12.4	11.8
Dicalcium phosphate	15.5	13.0	12.5
Salt	2.5	2.5	2.5
Mineral-vitamin premix *	5.0	5.0	5.0
dl-methionine	2.5	2.2	2.0
l-lysine	0.7	0.5	0.4
**Calculated composition**			
Metabolizable energy (kcal/kg)	2900	2950	3000
Crude protein (%)	22.75	21.00	19.00
Crude fat (%)	2.42	4.64	2.86
Crude fiber	2.95	3.01	3.26
Ash	4.35	4.29	4.02
Linoleic acid (%)	2.81	2.13	1.46
Calcium (%)	0.94	0.87	0.78
Available phosphorus (%)	0.42	0.38	0.35
Sodium (%)	0.19	0.17	0.15
Methionine (%)	0.61	0.58	0.53
Methionine + cysteine (%)	0.96	0.94	0.81
Total lysine	1.30	1.10	1.10

* Supplied per kg diet: Calcium pantothenate: 4 mg/g; Niacin: 15 mg/g; Vitamin B6: 13 mg/g; Cu: 3 mg/g; Zn: 15 mg/g; Mn: 20 mg/g; Fe: 10 mg/g; K: 0.3 mg/g; Vitamin A: 5000 IU/g; Vitamin D3: 500 IU/g; Vitamin E: 3 mg/g; Vitamin K3: 1.5 mg/g; Vitamin B2: 1 mg/g.

**Table 2:** Growth performance of broiler chickens fed different levels of summer savory extract (SSE).

Item	Level of SSE (mg/kg)					SME	*p*-Value	
	0	100	200	300	400		L	Q
**Feed intake (g/bird/d)**								
1–14 d	27.6	25.0	26.5	27.3	27.1	0.872	ns	ns
15–28 d	103.1	96.7	102.5	108.3	95.4	3.084	ns	*
29–42 d	151.7	140.2	151.9	144.7	141.4	4.084	ns	ns
1–42 d	94.2	87.3	93.6	93.4	88.0	1.682	ns	*
**Body weight gain (g/bird/d)**								
1–14 d	27.2	19.5	21.0	21.9	21.0	0.723	ns	ns
15–28 d	63.3	62.1	62.6	65.5	60.9	2.494	ns	ns
29–42 d	78.6	76.2	79.6	74.1	78.6	2.579	ns	ns
1–42 d	54.3	52.6	54.4	53.8	53.5	0.589	ns	ns
**FCR (g/g)**								
1–14 d	1.3	1.28	1.26	1.25	1.29	0.016	ns	ns
15–28 d	1.63	1.56	1.64	1.66	1.57	0.032	ns	ns
29–42 d	1.93	1.85	1.91	1.96	1.81	0.055	ns	ns
1–42 d	1.73	1.66	1.72	1.74	1.65	0.061	*	ns

SEM: standard error of the means; FCR: feed conversion ratio. Orthogonal contrasts: L = linear, Q = quadratic; ns: not significant; * *p* < 0.05.

**Table 3:** Blood biochemical parameters of broiler chickens fed different levels of summer savory extract (SSE).

SSE (mg/kg)	Blood Constituents (mg/dL)						
	Glucose	Uric Acid	Cholesterol	Triglycerides	HDL	LDL	ALP
0 (control)	145.1	1.9	192.3	155.9	63.7	97.5	661.2
100	154.1	0.7	208.8	158.3	102.2	74.9	480.9
200	111.9	0.9	168.4	69.3	77.4	77.1	610.8
300	105.6	0.9	133.3	70.7	72.9	46.3	590.3
400	173.1	0.7	166.8	122.2	83.5	58.9	547.9
SEM	9.67	0.12	14.40	16.50	4.74	13.19	28.15
*p*-Value							
L	*	ns	ns	ns	ns	ns	ns
Q	ns	**	**	*	**	*	**

SEM: standard error of the means; HDL: high density lipoprotein; LDL: low density lipoprotein; ALP: alkaline phosphatase. Orthogonal contrasts: L = linear, Q = quadratic; ns: not significant; * *p* < 0.05; ** *p* < 0.01.

**Table 4:** Immune response (log10) of broiler chickens fed different levels of summer savory extract (SSE).

SSE (mg/kg)	Immune Response Criteria						
	IgG (28 d)	IgG (42 d)	IgM (28 d)	IgM (42 d)	ND (42 d)	IBD (42 d)	IB (42 d)
0 (control)	1.3	2.8	2.0	2.3	4.7	3.8	1.8
100	1.3	3.8	1.7	3.7	4.7	3.8	2.7
200	2.0	3.3	1.3	3.7	7.3	3.8	2.5
300	2.3	4.0	1.3	3.7	6.7	3.6	2.8
400	1.0	3.3	1.7	3.0	6.7	3.8	2.8
SEM	0.316	0.466	0.342	0.342	0.428	0.148	0.199
*p*-Value							
L	*	ns	ns	*	**	ns	**
Q	*	ns	ns	*	*	ns	ns

SEM: standard error of the means; ND: Newcastle disease; IBD: infectious bursal disease; IB: infectious bronchitis. Orthogonal contrasts: L = linear, Q = quadratic; ns: not significant; *p < 0.05; ** *p* < 0.01.

**Table 5:** Ileal microflora of broiler chickens fed different levels of summer savory extract (SSE).

Level of SSE (mg/kg)	Bacteria Count (CFU/g)		
	*E. coli*	*Lactobacillus*	Lactobacilli/*E. coli*
0 (control)	17,384.0	5013.2	0.29
100	1631.8	3590.9	2.20
200	2881.8	4274.6	1.48
300	2360.2	2968.0	1.26
400	2620.2	3836.1	1.46
SEM	510.56	722.52	0.281
*p*-Value			
L	ns	ns	*
Q	**	ns	*

SEM: standard error of the means. Orthogonal contrasts: L = linear, Q = quadratic; ns: not significant; * *p* < 0.05; ** *p* < 0.01.
